# From two competing oscillators to one coupled-clock pacemaker cell system

**DOI:** 10.3389/fphys.2015.00028

**Published:** 2015-02-13

**Authors:** Yael Yaniv, Edward G. Lakatta, Victor A. Maltsev

**Affiliations:** ^1^Biomedical Engineering Faculty, Technion-IITHaifa, Israel; ^2^Laboratory of Cardiovascular Science, Biomedical Research Center, Intramural Research Program, National Institute on Aging, National Institutes of HealthBaltimore, MD, USA

**Keywords:** arrhythmias, coupled-clock pacemaker system, heart rate variability, mathematical modeling, sinoatrial node

## Abstract

At the beginning of this century, debates regarding “what are the main control mechanisms that ignite the action potential (AP) in heart pacemaker cells” dominated the electrophysiology field. The original theory which prevailed for over 50 years had advocated that the ensemble of surface membrane ion channels (i.e., “M-clock”) is sufficient to ignite rhythmic APs. However, more recent experimental evidence in a variety of mammals has shown that the sarcoplasmic reticulum (SR) acts as a “Ca^2+^-clock” rhythmically discharges diastolic local Ca^2+^ releases (LCRs) beneath the cell surface membrane. LCRs activate an inward current (likely that of the Na^+^/Ca^2+^ exchanger) that prompts the surface membrane “M-clock” to ignite an AP. Theoretical and experimental evidence has mounted to indicate that this clock “crosstalk” operates on a beat-to-beat basis and determines both the AP firing rate and rhythm. Our review is focused on the evolution of experimental definition and numerical modeling of the coupled-clock concept, on how mechanisms intrinsic to pacemaker cell determine both the heart rate and rhythm, and on future directions to develop further the coupled-clock pacemaker cell concept.

## Introduction

Under normal conditions, specialized, self-excitable pacemaker cells within the sinoatrial node (SAN) initiate the spontaneous action potentials (AP) that are conducted to the ventricle to entrain the rate and rhythm of ventricular myocytes contractions. The identities and the relative roles of the control mechanisms within heart pacemaker cells that ignite the AP have been debated for more than 50 years. The predominant theory later named “M-clock” advocated that the ensemble of surface membrane ion channels was sufficient to ignite spontaneous AP (reviewed in Maltsev et al., 2006). This concept promoted decades of extensive voltage-clamp studies that have led to identification of numerous ion-current components in pacemaker cells (reviewed in Wilders, 2007): L-type Ca^2+^ current (I_Ca,L_), outward-K^+^ currents (I_K_), etc. Importantly, some but not all investigators concluded that a hyperpolarization-activated “funny” current (I_f_), is the dominant M-clock current driving early diastolic depolarization. However, since the time of I_f_ discovery its major role in cardiac pacemaking was challenged (Vassalle, 1995) and further experimental and theoretical results led to an extensive debate on the role of I_f_ (reviewed in Maltsev and Lakatta, 2012). In the late 1980s, experimental evidence began to emerge on the role of Ca^2+^ in pacemaker function under normal physiologic conditions (for more details see Maltsev et al., 2006). Subsequent studies discovered that sarcoplasmic reticulum (SR), a major Ca^2+^ store, can spontaneously and rhythmically oscillate Ca^2+^ uptake and release forming additional oscillator mechanism in pacemaker cells, termed Ca^2+^-clock. Ca^2+^-clock together with the M-clock form the modern concept that coupled-clock pacemaker system controls the cardiac pacemaker cell function.

**Figure 1:**
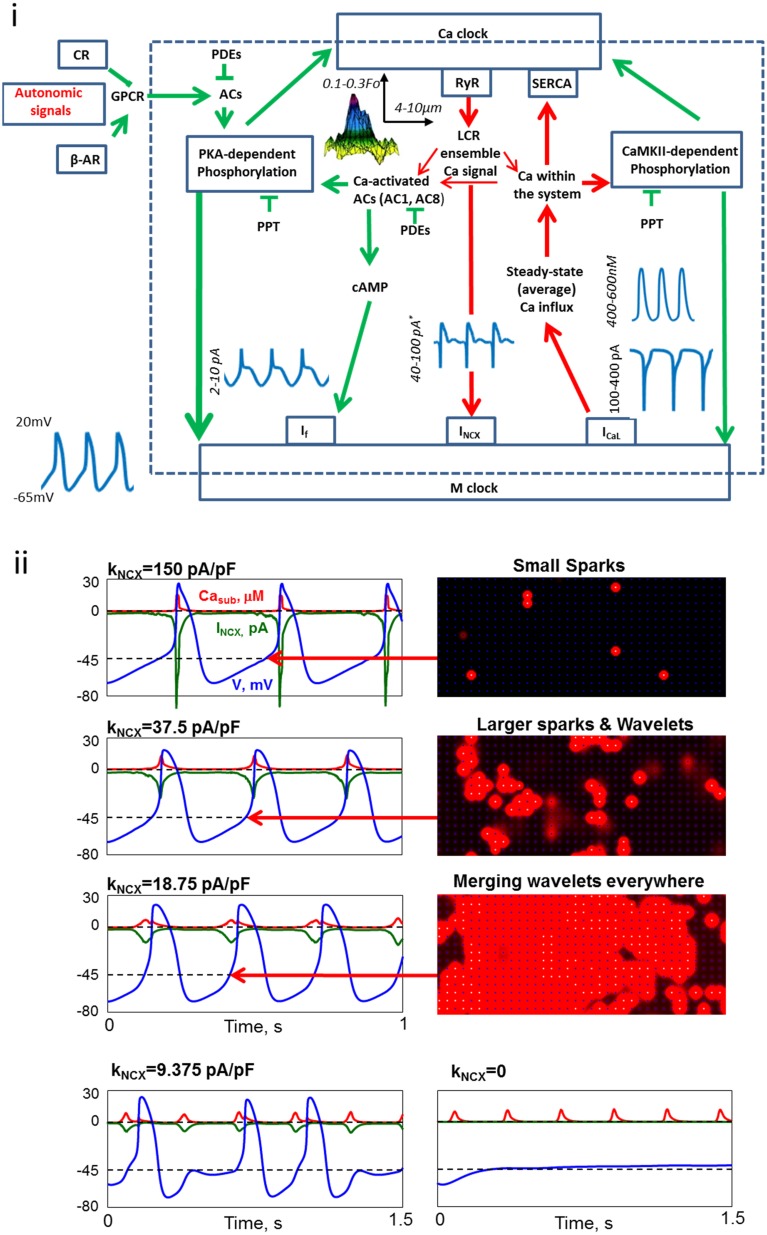
**Coupled-clock molecules and brain-heart signaling receptors that drive basal automaticity of SANC. (i)** The neurotransmitters noradrenaline (NE) and acetylcholine (ACh) released from sympathetic or parasympathetic nerve terminals bind to β-adrenergic receptors (β-AR) or cholinergic receptors (CR), respectively. Autonomic receptor signaling couples to G-proteins (GPCR) and leads to modulation of the same coupled-clock molecules that drive basal automaticity of SANC. Basal Ca^2+^-calmodulin activation of adenylyl cyclases (AC), which produce cAMP-PKA-dependent phosphorylation and calmodulin-dependent kinase II (CaMKII)-dependent phosphorylation signaling. cAMP positively shifts the f-channel activation curve. Phosphodiesterases (PDE) degrade cAMP production, while protein phosphatase (PPT) degrades phosphorylation activity. PKA and CaMKII signaling phosphorylate SR Ca^2+^ cycling proteins (RyR, phospholamban, which bind to and inhibit SERCA) and surface membrane ion channels.^*^The values are for I_NCX_ amplitude (within the cycle) achieved during systole, however the diastolic amplitude is almost an order of magnitude lower. **(ii)** Numerical model simulations of membrane potential (blue), I_NCX_ (green) and subspace Ca^2+^ (Ca_sub_, red) in response to reduction in NCX expression. As NCX expression becomes reduced the spread of Ca^2+^ release between RyRs via Ca^2+^ induced Ca^2+^ release is enhanced, resulting in a more effective activation of the remaining NCX molecules by LCRs. Further reduction in NCX uncoupled (partially or fully) LCR from AP generation. Specifically, NCX current becomes too small to depolarize the membrane and AP generation fail (modified from Maltsev et al., 2013).

To ignite an AP, the Ca^2+^-clock communicates with the M-clock via multiple Ca^2+^ and voltage-dependent mechanisms (discussed below). Nevertheless, one approach to gain further insights into the systems operation has been to artificially split the two clocks into two separate competing mechanisms (see for example Noble et al., 2010). A major consequence of such approach let to a continuing debate about which clock or pacemaker mechanism is dominant, and which one is minor (i.e., being a follower or entrained) (Lakatta and Difrancesco, 2009; Rosen et al., 2012). An alternative view is that both intracellular and sarcolemmal mechanisms are dynamically and synergistically coupled to each other (Figure 1), and the degree of the coupling determines the normal pacemaker function (Lakatta et al., 2010). This view, known as a coupled-clock theory, is based on the results of numerical modeling (Maltsev and Lakatta, 2009, 2010, 2013) and verified by experimental data (Yaniv et al., 2013a, 2014b). Therefore, a modern view on the cardiac pacemaker cell function is that neither clock is dominant; rather it is the coupled-clock system that controls the pacemaker cell AP firing rate and rhythm.

## Mechanisms intrinsic to pacemaker cell determine the coupled-clock rate

After more than 50 years of research it is apparent that the pacemaker function is orchestrated via intrinsic signaling mechanisms originating at multiple levels of organization, including subcellular (e.g., phosphorylation cascades, SR, mitochondria), cellular (i.e., surface membrane), hierarchical brain-heart signaling (i.e., neurotransmitter or hormonal stimulation of surface membrane receptors) and modulated by environmental mechanical, chemical and thermal factors (Figure 1). The SR rhythmically discharges local diastolic Ca^2+^ releases (LCRs) beneath the cell surface membrane; LCRs activate an inward Na^+^/Ca^2+^ exchange (NCX) current that prompts the surface membrane clock (M clock), an ensemble of sarcolemmal electrogenic molecules, to generate an AP. LCR Ca^2+^ signal is regulated not only by the SR Ca^2+^ pumping, which depends not only on SR proteins (phospholamban and RyR), and their phosphorylation status, but also by functions and phosphorylation status of M-clock proteins, e.g., L-type Ca^2+^ channels that regulate cell Ca^2+^ available for SR pumping, i.e., Ca^2+^-clock's substrate or “fuel.” LCR signals affect Ca^2+^ -dependent electrogenic processes (such as Na^+^/Ca^2+^ exchange) and voltage-dependent Ca^2+^ fluxes (such as via Ca^2+^-dependent inactivation of L-type Ca^2+^ channels). Therefore, the amplitude and phase of the LCR Ca^2+^ signal sensed by M-clock proteins reports the degree of synchronization and coupling of pacemaker mechanisms of both clocks, i.e., a stronger, more synchronized, and earlier LCR signal to M-clock proteins reports more efficient clock coupling that results in further shortening of the AP-beating interval (BI).

The coupled-clock theory predicts that extremely complex crosstalk between the two clocks via signaling pathways can amplify each other via secondary (indirect) mechanisms (reviewed in Maltsev and Lakatta, 2012), e.g., the crosstalk determines cell Ca^2+^ which, in turn, activates calmodulin-adenylyl cyclase (AC)-dependent protein kinase A (PKA) and Ca^2+^/calmodulin-dependent protein kinase II (CaMKII) (Mattick et al., 2007; Younes et al., 2008; Yaniv et al., 2013b). These phosphorylation signaling cascades act on both SR (phospholamban and RyR) and M-clock proteins (such as L type Ca^2+^ channels and K^+^ channels). Numerical model simulations predict that the diastolic LCR signal is also regulated both by the level of Ca^2+^ cycling, and by the phosphorylation states of coupled-clock proteins (Maltsev and Lakatta, 2009; Yaniv et al., 2013a; Stern et al., 2014). Indeed, the LCR period (i.e., the time period of an LCR occurrence following the prior AP) reports the degree of synchronization of the coupled-clock mechanisms (Monfredi et al., 2013; Yaniv et al., 2013a, 2014b). Thus, during higher degrees of clock coupling AP BI and LCR period are shorter and vice versa. LCRs affect Ca^2+^ dependent mechanisms, specifically NCX, whereas the M-clock effects Ca^2+^ clock primarily via I_Ca,L_. Phosphorylation signaling acts on both clocks and a decrease in its level is correlated with a decrease in the degree of synchronization of the coupled-clock mechanisms (Yaniv et al., 2014b). Numerical evidence has shown the essential roles of both mechanisms to couple clock functions (see below).

Although majority of the original experiments supporting the coupled-clock concept were performed in rabbit pacemaker cells, recent experimental results from mouse genetic models have clarified the role of many coupled-clock components: NCX (Groenke et al., 2013; Herrmann et al., 2013), I_f_(Ludwig et al., 1998; Stieber et al., 2004; Herrmann et al., 2007), T-type channels (Mesirca et al., 2014), G protein signaling (Yang et al., 2010; Wydeven et al., 2014), Cav1.3 (Christel et al., 2012), CaMKII activity (Zhang et al., 2005; Gao et al., 2011) and ankyrin-B function (Le Scouarnec et al., 2008). In this regard mice pacemaker cell model provides evidence for the role of TRP channels and IP3 receptors; TRPM4 channels conduct both Na^+^ and K^+^, but does not conduct Ca^2+^. TRPM has been recognized as the Ca^2+^-activated nonselective cation channel (Demion et al., 2007) and its role in modulating AP firing rate has been shown recently (Hof et al., 2013). Specifically, TRPM7 has been shown as a dominant channel-kinase that influences diastolic membrane depolarization (Sah et al., 2013). 1,4,5-trisphosphate (IP3) receptors exist in mice pacemaker and can release Ca^2+^ from the SR contributing to the intracellular Ca^2+^ that couples both clocks (Ju et al., 2011, 2012). Note that the relevant of studies in mice to other species with much lower hear rate has to be proven.

## Mechanisms intrinsic to pacemaker cells control AP firing rate and rhythm

**Figure 2:**
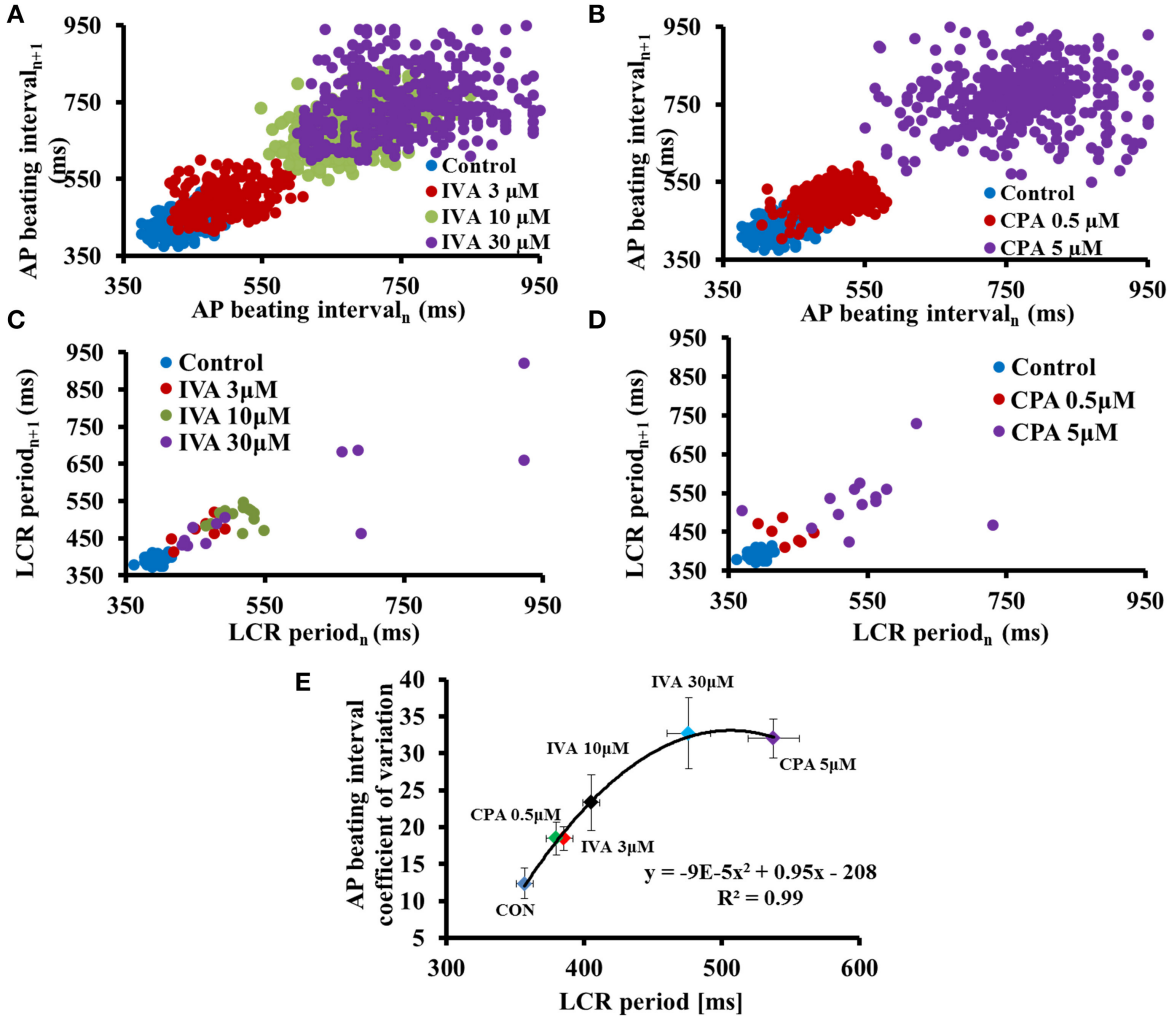
**Reduced synchronization of coupled-clock mechanisms prolongs AP beating interval variability and LCR period variability**. To unravel clock-crosstalk effects on AP BIV and LCR period variability, clock function was perturbed by directly inhibiting either the M or Ca^2+^-clock. To inhibit the M-clock, a range of concentrations of ivabradine (IVA), an I_f_ inhibitor were employed. To inhibit the Ca^2+^-clock, a range of concentrations of cyclopiazonic acid (CPA), a SR Ca^2+^ pump inhibitor were employed. Poincaré plots of the beating interval in control and in response to **(A)** IVA or **(B)** CPA. Poincaré plots of LCR period in control and in response to **(C)** IVA or **(D)** CPA. **(E)** The relationship between the average AP BIV, quantified by coefficient of variation to the LCR period prior to and in response to different concentrations of either IVA or CPA (modified from Yaniv et al., 2014b).

The spontaneous AP BI of single isolated pacemaker cells and SAN tissue are roughly periodic, i.e., this period varies on a beat-to-beat basis (Verheijck et al., 1998; Rocchetti et al., 2000; Zaza and Lombardi, 2001; Monfredi et al., 2011; Papaioannou et al., 2013; Yaniv et al., 2014a,b). Recent experimental evidence shows that the degree of clock coupling determines not only the average pacemaker cell AP BI, but also the AP beating interval variability (BIV) (Yaniv et al., 2014b) (Figure 2). LCR periods vary among individual LCRs occurring within each spontaneous AP cycle and, similar to AP BI variability, among different cycles (Monfredi et al., 2013; Stern et al., 2014; Yaniv et al., 2014b). The ensemble LCR period and size report the extent of synchronization of the coupled-clock mechanisms. Indeed, the variability in the average LCR period in each cycle is correlated with the variability of the AP BI (Monfredi et al., 2013; Yaniv et al., 2014b) (Figure 2) and beat-to-beat variation in periodicity of LCRs is associated with intrinsic variations of spontaneous AP BI (Monfredi et al., 2013). Based on the coupled-clock theory, the stochasticity of LCR Ca^2+^ signal depends on stochastic RyR activation (Stern et al., 2014) and the cell Ca^2+^ balance that in turn is determined, in part, by stochastic sarcolemmal ion channel openings and closings. The occurrence of an AP synchronizes global stochastic RyR activation, and therefore synchronizes subsequent generation of LCRs by the RyRs during the diastolic depolarization phase. The amplitude of LCR Ca^2+^ signal to M-clock proteins reports the efficiency of clock coupling, i.e., a weaker LCR signal to M-clock proteins reports less-efficient clock coupling. At steady state, increase in LCR variability is also linked to reduced peak ensemble LCR Ca^2+^ signal amplitude that occurs later in diastole (i.e., prolonged next AP ignition). Therefore, the extent to which intrinsic clock mechanisms regulates the coupled-clock determines both the steady state BI and BIV in isolated pacemaker cells.

## Brain modulation of the intrinsic mechanisms to pacemaker cell

The brain imparts flexibility to intrinsic clock mechanisms by concomitant activation of two types of receptors: β-adrenergic receptors (β-AR) that increases the heart rate and cholinergic receptors (CR) that decreases the heart rate (Figure 1). In humans, change in receptor activation can change the heart rate from 60 to 240 bpm. Receptor stimulation within pacemaker cells couples the signaling of G-proteins to AC (likely type 5 or 6), leading to activation or suppression of PKA and CaMKII- dependent phosphorylation signaling to key functional proteins of both clocks that regulate pacemaker cell automaticity. Therefore, both brain-heart signaling and intrinsic-pacemaker cell mechanisms signal to the very same nodes (coupling factors, such as PKA and CaMKII, Figure 1) of the coupled-clock system

β-AR stimulation in single pacemaker cell not only markedly decreases the average AP BI, but also decreases the AP BIV indices (Zaza et al., 1996; Yaniv et al., 2014a) and increases the likelihood that pacemaker cell BIs exhibit fractal-like behavior (Yaniv et al., 2014a). β-AR stimulation increases the efficiency of the coupled-clock system (Yaniv et al., 2014a). A reduction in LCR variability is associated with increased peak ensemble LCR Ca^2+^ signal that occurs early in diastole (Monfredi et al., 2012; Yaniv et al., 2014b). β-AR stimulation decreases the beating-interval entropy, which in isolated pacemaker cells is within a range that has been documented in random systems. Therefore, β-AR stimulation confers beating interval complexity. CR stimulation, in contrast, not only markedly increases both the average AP BI and AP BIV indices of single isolated pacemaker cells, but also impairs beating interval complexity (Rocchetti et al., 2000; Zaza and Lombardi, 2001; Yaniv et al., 2014a). Therefore, CR stimulation reduces the efficiency of the clocks coupling (Yaniv et al., 2014a).

## The coupled clock system operates on a beat-to-beat basis

While a mutual entrainment exists between the M and Ca^2+^-clocks, it was not known if this entrainment happens on a beat-to-beat basis. Patch clamp experiments in single SAN cells (SANC) appeared to show a minor role of Ca^2+^ dynamics in SANC function (Himeno et al., 2011). The spontaneous AP rate was little changed when BAPTA, a Ca^2+^ chelator, was acutely infused via a patch pipette into SANC. These results, however, were later refuted on technical grounds (Maltsev et al., 2011b), taking into account that whole cell configuration generates artificial leak currents that substitute the pacemaker currents.

In contrast to these results, three sets of experiments that rapidly perturb the Ca^2+^-clock in intact SANC have demonstrated the time-dependent beat-to-beat mutual entrainment between the two clocks. In the first experiment set single isolated rabbit SANC were loaded with a caged Ca^2+^ buffer, NP-EGTA, which induced an increase in AP BI and markedly suppressed LCR Ca^2+^ signals and uncoupled them from AP generation (Yaniv et al., 2011). Flash photolysis released Ca^2+^ from the caged compound, immediately restored Ca^2+^ dynamics and within the same AP cycle. In the second experiment set low concentrations of caffeine (2-4 mM) were rapidly applied to single isolated rabbit SANC (Yaniv et al., 2013c). Caffeine induced immediate Ca^2+^ release from the SR and immediately reduced the AP cycle. Lastly, in each given cycle the phase of the entire ensemble LCR signal (i.e. the average LCR period) is linked to that length of that cycle (Monfredi et al., 2013). Therefore, mutual clock entrainment exists on a beat-to-beat basis.

## Numerical modeling: from one clock to one coupled system

The shift from numerical models that describe only the M-clock to the new paradigm of the coupled-clock system occurred in several stages, as new data became available and new respective models were generated. The first model that attempted to explore the importance of Ca^2+^ levels in sub-membrane space with respect to M-clock molecules (specifically the NCX) was formulated by Kurata (Kurata et al., 2002). While this modeling approach reproduced, in part, bradycardic effects of intracellular Ca^2+^ buffering reported earlier in experimental studies (Vinogradova et al., 2000), it remained essentially naive and did not embrace a numerical mechanism of Ca^2+^ “clocking.”

The coupled-clock mechanism was established in 2009 (Maltsev and Lakatta, 2009) by a detailed sensitivity analysis of a new pacemaker cell model originated from Kurata et al. model (Kurata et al., 2002). This new model (often referred to as Maltsev-Lakatta model or ML model) included, in addition to the formulations of the M-clock molecules, new formulations of the SR function, predicting oscillatory LCR ensemble Ca^2+^ signals, driven by SR Ca^2+^ pumping and Ca^2+^ release kinetics. The two clocks are coupled in the ML model via multiple coupling factors, such as Ca^2+^, cAMP, PKA, and CaMKII. Therefore, the SR Ca^2+^-clock not only modulates the M-clock, but the M-clock, in turn, also affects Ca^2+^-clock.

This coupled-clock model made important predictions that prompted further studies:

- Importance of SR Ca^2+^ refilling kinetics for AP firing rate (confirmed experimentally in Vinogradova et al., 2010)
- Both “biophysical” and “biochemical” entrainments are required to explain complex effects of clock's-specific perturbations (Yaniv et al., 2013a), e.g., by either ivabradine, a specific I_f_ blocker or cyclopiazonic acid, a specific SR Ca^2+^-ATPase (SERCA) pump blocker. It was shown that a direct perturbation of one clock inevitably affects the other due to subsequent indirect effects, resulting in mutual entrainment, i.e., clocks coupling, predicted by the theory.
- The entire range of physiological chronotropic modulation of SANC by activation of β-AR or CR can be achieved in simulations of the ML model only when their effect on both sarcolemmal ion channels and SR Ca^2+^ pumping capability are taking into account (Maltsev and Lakatta, 2010).

Of note, other numerical models with unbalanced mutual entrainment between the clocks have been developed (Zhang et al., 2000; Butters et al., 2010; Inada et al., 2014). Specifically, recent numerical model has applied to explain the relationship between heart rate and rhythm (Monfredi et al., 2014). Although these models have some merit and can explain some experimental results, the real test or value of numerical models is to reproduce the experimental data of mutual entrainment on a beat-to-beat basis, which these simplistic models cannot achieve.

Mutual entrainment of the Ca^2+^ and M-clock exists on a beat-to-beat basis (see above). Numerical simulations, using a modified ML “coupled-clock” model, faithfully reproduced experimentally reported prolongation of the AP BI and associated dys-rhythmic spontaneous beating in the presence of cytosolic Ca^2+^ buffering (Yaniv et al., 2013c). However, three contemporary numerical models (Kurata et al., 2002; Severi et al., 2012) and the original ML model (Maltsev and Lakatta, 2009), failed to reproduce the effects of severe and acute perturbations of the system, e.g., the transient reduction in AP BI induced by both caffeine and flash-induced Ca^2+^ release (Yaniv et al., 2013c).

The modified ML model provided new insights into the nature of beat-to-beat clock entrainment (Yaniv et al., 2013c): (i) The major mechanisms that couple the beat-to-beat changes in Ca^2+^-clock to M-clock mechanisms is LCR-activation of the NCX current. (ii) The systems has a “memory” for several beats: after flash-induced Ca^2+^ release the temporal rate increase are linked to changes in Ca^2+^ available for pumping into the SR that ultimately results in a temporal increase in diastolic NCX current driving the AP firing rate increase.

Recognition of the limitations of the traditional common-pool model approach have led to novel pacemaker cell models featuring local Ca^2+^ control mechanisms (for review see Maltsev et al., 2014). Thus, newer local control models are more accurate vs. old common pool models: the scale of amplitudes for Ca^2+^ dynamics attained locally is higher by as much as two orders of magnitude vs. that predicted by the old models. The common pool models also lack crucial mechanisms of Ca^2+^ dynamics, such as diffusion-reaction for the Ca^2+^ release, local Ca^2+^ pumping and local NCX activity. The first model (Maltsev et al., 2011a) generated LCRs via stochastic recruitment of the neighboring CRUs. This model was later updated to include LCR regulation by local interactions with M-clock driving by NCX (Maltsev et al., 2013). The model predicted that when the RyR sensitivity is very high or the NCX density is low, synchronization between the clocks is lost, leading to dysrhythmic AP BI (Maltsev et al., 2013). The most recent and advanced formulations of local Ca^2+^ mechanisms in pacemaker cells include stochastic gating of individual RyR and L-type Ca^2+^ channels in Ca^2+^ diffusion and buffering in 3 dimensions (Stern et al., 2014). The model succeeded in reproducing observed propagating local Ca^2+^ releases and realistic pacemaker rates only when RyR locations were assigned taking into account irregular, hierarchical distribution of RyR clusters (small and large) observed in 3D confocal scan sections of immunofluorescence staining.

A new generation of model featuring local Ca^2+^ dynamics within a coupled-clock system is being developed and will provide novel insights into pacemaker cell mechanisms. A “multi-scale” modeling has been put forward by James Weiss group that modeled Ca^2+^ dynamics in cardiac cells (Qu et al., 2011). This approach develops formulations for Ca^2+^ dynamics at each level or scale of integration. This, in turn, represents a substantial challenge and requires a detailed knowledge of the previous layer to avoid simply phenomenological or arbitrary descriptions. In this regard, the recent Stern et al. model (2014), describing states of all individual RyR and L-type Ca^2+^ channel will be helpful to approach the next level integration at the whole cell Ca^2+^ dynamics.

## Summary

Mechanisms intrinsic to pacemaker cells and their modulation by the brain-heart receptor signaling determine both the heart rate and heart rate variability. Crosstalk exists between M and Ca^2+^-clock and the tightness of this crosstalk, informed by the LCR period, determines the rate and rhythm of spontaneous AP generation. Indeed, both theoretical and experimental evidence has mounted to indicate that this clock “crosstalk” operates on a beat-to-beat basis.

In the level of pacemaker cells, future experiments are needed to quantify beat-to-beat regulation of cAMP/PKA signaling that drives clock coupling in order to speculate whether they take part in the mutual entrainment between the clocks in a beat-to-beat basis. Moreover, the extent to which reduction in synchronization of intrinsic clock periods within pacemaker cells is associated with cardiac diseases and aging awaits further elucidation. Finally, novel mathematical models that quantify not only the average AP firing, but also determine its rhythm await further development.

Similar contribution of coupled-clock mechanisms to membrane firing rate and rhythm can exist in other heart tissues. In this regard, crosstalk between Ca^2+^ leak from the SR and NCX current can trigger an arrhythmia in atrial fibrillation patients (Lakatta and Guarnieri, 1993; Voigt et al., 2014). Future work is needed to determine if pacemaking-like behavior exist in atrial cell during normal and abnormal conditions.

### Conflict of interest statement

The authors declare that the research was conducted in the absence of any commercial or financial relationships that could be construed as a potential conflict of interest.
